# From Self-Doubt to Pride: Understanding the Empowering Effects of Delivering School-Based Wellness Programmes for Emerging Adult Facilitators—A Qualitative Study

**DOI:** 10.3390/ijerph19148421

**Published:** 2022-07-10

**Authors:** Galia Ankori, Dana Tzabari, Tamar Hager, Moria Golan

**Affiliations:** 1 Faculty of Social Sciences and Humanities, Tel-Hai College, Upper Galilee 1220800, Israel; tamar.hager@gmail.com; 2 Child and Adolescence Mental Health Clinic of Maccabi Health Services, Sderot Binyamin 21, Netanya 4231111, Israel; 3 Department of Nutritional Sciences, Tel-Hai College, Upper Galilee 1220800, Israel; danatzabari91@gmail.com (D.T.); moria.golan@mail.huji.ac.il (M.G.); 4 Shahaf, Community Based Facility for Body Image and Eating, Ganey Hadar 7683000, Israel

**Keywords:** prevention programs, program’s facilitators, emerging adulthood, qualitative, pride, fear, self-determination theory

## Abstract

Ample literature exists on the impact of prevention programmes on their target audience, while much less is known about how delivering such programmes influences their facilitators. Even less literature exists on the emotional and social processes that form this potential impact on facilitators. The current study analysed qualitative in-depth, non-structured interviews, as well as written essays provided by 33 student-facilitators who delivered the “Favoring Myself” programme in Israel during 2019–2021. This school-based wellness programme comprised 10 weekly, 90 min sessions on self-care behaviours, media literacy, self-esteem, and positive body image, which are well-known protective factors against risky behaviours. A thematic analysis was applied to explore the main themes in the collected data. An interesting affective transformation from self-doubt to pride in themselves emerged as a shared experience of these young facilitators. Facilitators related their ability to facilitate the programme, as well as to undergo an individual maturation and empowerment experience, to certain components of the programme itself, such as the preparatory course, individual supervision, and the peer-group experience. This shift from doubt to pride is discussed using two frameworks—a theoretical discourse of emerging adulthood as a developmental stage, and the self-determination theory.

## 1. Introduction

The impact of prevention programmes on their audiences is well-documented [1,2]. Nevertheless, a literature review yielded only few studies on the impact of such programmes on their facilitators. The current study aims to fill this gap in the literature by qualitatively exploring the emotional impact of the prevention and wellness programme “Favoring Myself” on its facilitators, students in their emerging adulthood, complementing a previous quantitative study [3].

Prevention science is based on the identification of predictors, risks, and protective factors for problem behaviours [4]. A large body of scientific evidence shows that risky behaviours can be prevented, using three types of prevention programmes: universal programmes, targeting all children without regard to level of risk exposure; selective programmes, which focus on children who have been exposed to elevated levels of risk but who do not yet manifest behavioural health problems; and indicated programmes, which focus on youth who evidence early symptoms of behavioural health [5]. Factors that may influence the effectiveness of prevention programmes include programme format, participant risk status, participants’ age, type of intervention, and number of sessions [6,7]. Although there have been some reports on the efficacy of after-school prevention programmes [8,9], most preventive programmes are school-based, and their impact has been described in numerous publications [10]. Skryabina et al. [11] demonstrated a significant improvement when their programme was delivered by external facilitators, versus no change when the school’s team delivered the programme. Nevertheless, Yager et al. [10] found that the presence of the class’s teacher during the delivery of the programme contributed much to the success of their prevention program. Based on these insights from the literature, the fourth author developed “Favoring Myself”, an interactive, school-based, universal program that has been integrated into school curricula in Israel as part of a mandatory class on life skills. “Favoring Myself” was reported to successfully promote positive self-esteem and body image among pre-adolescents and adolescents [12]. A change in these variables is most important, since self-esteem and body image are negatively correlated with risky behaviours [13,14].

The programme facilitators were college students in their early twenties, majoring in Education or Nutritional Sciences. To become a facilitator, participants were interviewed by a social worker with expertise in group work, who evaluated their motivation to take part, emotional maturity, sense of competence, and ability for teamwork. Facilitators were then assigned to establish joint facilitation teams, with each student co-leading a group with a member from the other department in the college. Programme facilitators participated in a preparatory course that included simulations in which they practiced facilitating skills and experienced the same processes that they learnt to facilitate. In addition, facilitators participated in a weekly group supervision session with a social worker. Individual supervisions were held for each facilitator once every three weeks, and once every four weeks they were supervised together with their co-leaders.

Interest in this programme’s facilitators’ side of the story unfolded from the unintended accumulation of comments made by facilitators regarding their emotional experiences. Many facilitators shared with their peers and supervisors that they experienced concerns or fears before they began facilitating the program. Most of them were surprised by the impact of the process, and the empowerment and emotional growth they sensed at the end. One student-facilitator, for example, who at the onset of the training programme was unable to introduce herself to her peers, hardly murmuring her name, ended the program with the ability to give a successful presentation in front of the group. She later said that what helped her was that she realised she does not have to be perfect, and that it is okay to be embarrassed, and not all-knowing. She gradually began enjoying the experience of facilitating the children’s groups and felt immensely proud of herself. Other facilitators talked about feeling proud about taking better care of themselves, for example, by decreasing their alcohol use and unsafe sexual behaviours.

Realising that the facilitators began the process with significant concerns and went through a process they considered meaningful, the authors became interested in exploring the emotional impact of facilitating the programme on its facilitators. The authors hoped that insights from the study would assist developers of other prevention programs in designing appropriate training and ongoing supervision for their facilitators.

*The current study explored facilitators’ emotional experiences prior to and at the end of facilitating the programme. The literature review includes three topics: 1. Publications discussing how facilitating influences the facilitators; 2. Emerging adulthood as a developmental stage, particularly in terms of affective processes; and 3. Self-determination theory as a way to conceptualise internal motivational processes that might have helped students endure the many challenges they faced while facilitating the programme.*

### 1.1. Experiences of Facilitators and Mentors 

A thorough literature review yielded scarce literature on the experiences of facilitators and mentors. This literature demonstrates how mentoring or delivering prevention programmes improves self-care, abilities, confidence, and relationships. Jennings et al. explored the impact of leading a school-based sex education programme on the facilitators [15]. They found that facilitators reduced their own risky sexual behaviours. In these authors’ view, this resulted from developing the confidence to avoid high-risk measures. Crisp et al. described a stress-prevention programme, whose facilitators reported an increase in their confidence, as well as social connectedness and belonging to their university community [16]. Students who mentored youth were found to further develop their values, skills, and relationships [17]. These studies are important and relevant to the experiences of facilitators, but none of them explored emotional experiences in relation to and during the facilitation process.

Several researchers have been concerned with this lack of research and have recommended further evaluation of personal changes that facilitators experience as they take on these leadership roles [13,18,19,20]. The current study addressed this call by eliciting impressions, narratives, and insights from facilitators and exploring how these are related to their developmental stage in life—emerging adulthood. 

### 1.2. Emerging Adulthood—Social and Emotional Issues

Emerging adulthood is a developmental stage lasting between the ages of 18 and 26. These years are considered volitional, in that they offer opportunities for identity exploration in the areas of love, work, and worldviews [21]. During this period, individuals begin to develop qualities necessary for becoming self-sufficient, engage in mature and committed relationships, assume more responsibilities, and obtain a level of education that sets the foundation for work. New capacities develop that promote emerging adults’ abilities to reflect on their environment as well as on their internal states. They show improvement in regulating their emotions, and in using problem-solving skills to effectively compromise, which is considered important for the development of meaningful social and personal interactions [22]. 

Arnett, who had introduced the concept at the beginning of the millennium, writes about emerging adults as experiencing both excitement and uneasiness, wide-open possibility and confusion, and new freedoms as well as new fears [23]. Arnett [24] proposed five distinct dimensions related to psychological states that characterise early adulthood: 1. Identity exploration—young people explore different ways of living; 2. Experimentation—an optimistic time with many opportunities; 3. Negativity—feeling overwhelmed and unsettled; 4. Feeling in-between—feeling no longer adolescent but not yet fully adult; and 5. Self-focus—autonomy and personal freedom. 

Along this line of considering both resources and challenges in this period, when exploring the affective experience of emerging adults, it would be appropriate to pay attention to both positive and negative emotions. The diversity of opportunities is unsettling for many people [24,25], and may be a source of social and developmental vulnerabilities [26]. Volková and Dušková studied fears, defined as normal responses to danger, in emerging adults [27]. They found fears related to autonomy and identity, specifically in relation to school performance and other potential failures. Their participants reported fears of incompetence, uncertainty regarding managing situations, fear of not learning from mistakes, not being able to take action, and not fulfilling their life goals. In another study, college students’ specific fears of speaking in public included various scenarios; for example, not speaking fluently, not maintaining eye-contact, being restless, not being well-prepared, facing unfavourable audience responses, and encountering unexpected events [28]. In addition to these normative fears, some emerging adults experience a more profound identity crisis that may lead to anxieties [24,25], depressive symptoms [26], and impaired self-care behaviours [29,30]. 

Self-doubt is also considered a negative affect prevalent in emerging adulthood [27]. Self-doubt represents a sense of uncertainty about one’s important abilities. Most people experience self-doubt at times without negative consequences, but chronic self-doubt could present a threat to the need for competence, viewed as significantly related to psychological well-being. Substantial evidence suggests that chronic self-doubt is associated with low self-esteem and a sense of unworthiness [31]. Nevertheless, those researchers claim that some people perceive self-doubts as an important motivator, and that the perceptions regarding self-doubt are culturally dependent. 

Arnett’s dimensions of ‘self-focus’ and ‘experimentation’ [24] are considered potentially positive. Kuwabara et al. described emerging adults as optimistic [26]. Conroy et al. studied pride, a self-conscious emotion, that reflects the status of the self in relationships [32]; it is evoked in contexts that enhance a person’s social status and prestige, which is potentially quite common when young people pursue achievements. Experiencing pride is important since it predicts resilience, life satisfaction, and the ability to persevere despite an absence of external rewards [33]. Another study showed that pride positively correlates with students’ self-efficacy and adequacy, performance, overall achievement, and intrinsic motivation [34]. Since motivational factors, particularly internal motivation, seem to be salient in the lives of emerging adults, the self-determination theory can be used to contribute to this discussion. 

### 1.3. Self-Determination Theory 

Self-determination theory [35,36] is a theory of human motivation, emotion, and development that focuses on factors that either facilitate or forestall the assimilative and growth-oriented processes in people. According to this theory, three psychological needs motivate people: relatedness, competence, and autonomy. Relatedness means that human beings have the need to be part of a community, in which they feel protected and worthy of love. Competence is represented by a person’s wish to experience oneself as capable of achieving goals, actualising plans, and being efficient. Autonomy is the need to take self-directed actions, exercise the freedom of choice, and feel that one’s actions meet various needs, including self-fulfilment and pleasure [37].

## 2. Materials and Methods

Based on the literature presented, the authors decided to conduct an exploratory qualitative study that would elicit impressions, narratives, and insights from facilitators of “Favoring Myself”. The authors were curious to find out whether previous findings of improvements in self-care, confidence, and relationships during the facilitation process would arise in this programme’s facilitators’ experiences as well. In addition, the authors were interested to find out if developmental concepts as well as insights from self-determination theory would indeed shed light on the facilitators’ experiences. Since the study was exploratory, the authors remained open to the possibility of new and surprising themes being revealed in the facilitators’ narratives. 

### 2.1. Study Design 

The qualitative research presented in this article is part of a controlled trial, designed to evaluate the impact of facilitating “Favoring Myself”, a school-based wellness programme, on its facilitators. This paper describes the outcome from two measures: in-depth, semi-structured interviews with these facilitators, as well as themes that emerged in their final academic essays.

### 2.2. Research Approach 

Choosing a qualitative approach stemmed from the search for an interpretive, subjective manner of studying people’s own perceptions of events to understand and interpret reality from their points of view, highlight and explain life experiences, and give them meaning. Qualitative research allows us to deeply explore different perspectives and to discover the complexities of situations through a holistic framework [38,39,40]. Qualitative methods are more appropriate for an understanding of reality and knowledge as constructed, rather than as ‘truth’ [41]. In addition, this method is considered appropriate for data collected in naturalistic settings, like ours, in which the researchers are also active members of the system they study [42]. 

### 2.3. Participants

Participants of this study were the facilitators of “Favoring Myself”, students enrolled in a bachelor’s degree course at Tel-Hai College. All 33 student-facilitators (mean age 26.0 ± 1.4) who delivered the program during the 2019–2021 session agreed to participate in this qualitative study. Of these, 20 student-facilitators agreed to be interviewed, and 30 student-facilitators shared their final essays with the researchers. The study population’s personal and sociodemographic characteristics are displayed in Table 1. 

### 2.4. Data Collection 

Data for this study was collected from in-depth interviews as well as essays that the student-facilitators submitted at the end of their academic course.

#### 2.4.1. Interviews

A month after concluding the programme (including the preparatory course and the facilitating process in the schools), one-time semi-structured interviews with the facilitators were conducted. All interviews were conducted one-on-one by the second author. Interviews lasted approximately 45–50 min and were audio-recorded and then transcribed by the second author. The opening inquiry encouraged the participants to talk about their experience facilitating “Favoring Myself”. Participants then talked freely, with intermediate loosely structured prompts, meant to ensure that participants would touch upon core aspects, including the co-facilitating experience (e.g., How was working with a co-leader for you? How did you divide responsibilities and roles between the two of you?), the training process (e.g., How was the preparatory course for you?), relationships with the peer group (e.g., How did you experience the peer-group supervision sessions?), etc. (see Supplementary Material S1). 

#### 2.4.2. Final Essays

As this study was part of an academic course, student-facilitators were assigned a final essay. The instruction for this essay included a request to describe the personal experience of the facilitator with respect to several aspects, such as their relationships with the class they facilitated, their co-leading experience, the process of receiving personal supervision, etc. (see Supplementary Material S2).

### 2.5. Ethical Considerations

This study was approved by the Institutional Review Board and was carried out in accordance with the standards of Good Clinical Practice. It had been registered with ‘Contact ClinicalTrials.gov PRS’ (Trial registration: NCT03882242). Written informed consent was obtained from all participants. All names and personal details mentioned in the current paper were changed, to protect participants’ privacy.

### 2.6. Data Analysis

Data from both interviews and academic papers were analysed using the thematic analysis approach. Since qualitative interpretation requires multiple perspectives, the initial stage was performed independently by two of the researchers as well as an outside colleague. To reduce the potential for interpretation bias, each of the three peers read through all interviews and final essays several times and took notes, identifying recurring themes. One of the authors and the outside colleague began with the interviews, while the other author began with the essays. In this way the advantages of using different types of data were maximised. Stories of different participants which seemed to have common motives were connected, in order to represent processes in the internal worlds of participants [43] and understand the combined meanings of these texts [44,45]. Only then the authors met and discussed their coding and interpretation of the findings in order to formulate a diverse, yet mutual understanding of the findings.

## 3. Results

The results of the thematic analysis are organised into three categories: (1) participants’ concerns prior to facilitating, (2) participants’ pride after completing the facilitation period, and (3) factors that supported the facilitators. 

### 3.1. Participants’ Concerns Prior to Facilitating

Many facilitators had significant fears before they embarked on the facilitation process. As shall be seen, some were worried about the actual facilitator role, while others dealt with fears regarding working closely with a co-leader. Some facilitators were concerned regarding the individual or peer-group supervision. Anticipating her first facilitating experience, Lea said:

*This was my first time working with children. I was afraid about children disrespecting me and the contents I teach. I was worried about them making fun of me. What if they complain about being bored?!*

Lea’s concerns were quite common. Many future facilitators talked about stage fright, social anxiety, and other issues pertaining to the wish to perform well and the fear of failure. Some were worried they would attempt to placate the class and not allow themselves to exercise authority when needed. 

Tania connected her own self-doubts to her experiences at school as a child:

*My personal process began with one big word—Fear. I felt I was about to jump into deep frozen water at the beginning of a very cold winter. I decided to begin all of this because something deep inside me explained to Tania, the girl in me, that this was for her, that I have a lesson to learn here. To get something for my future, another tool for “down the road”. I hardly slept the night before the first facilitation, I woke up as if a war had begun, and there I was, entering school, the place most hated by Tania the girl. I think about that moment now, and it makes my skin crawl, especially when this thought pops up all the time: “the cute fat girl”.*

An interesting concern was raised by Tami who talked about her fear that teaching about self-esteem and body image, while she herself was still struggling with those issues, would not be authentic. She viewed children as a ‘radar’ that would sense and mark her lack of authenticity. Another student felt particularly self-conscious when presenting in front of the other students in the preparatory course. She was frustrated because she thought she was the only one experiencing this lack of confidence. She was convinced that her peers would notice her ‘weird behaviours’.

Regarding co-leading, some facilitators unfavourably compared themselves to their more experienced co-leader, whom they saw as flawless, while others were afraid to overrule a less confident co-leader. One of them, who felt particularly critical of her co-leader, was horrified she might replicate the hurtful confrontational style she had experienced in her family if she tried to pronounce her disagreement. 

In relation to their participation in the peer-group supervision, facilitators were afraid they may feel as ‘outcasts’, face judgement by others, or somehow damage their image in the eyes of others of even in their own eyes:

*Talking about weaknesses is a big challenge, on the one hand I do want to open up and I feel mature and ready to move forward, on the other hand it is important for me to maintain the image of the strong and “perfect” girl.*

In summary, it seems that facilitators had a genuine yearning to be authentic and give something meaningful to the children as well as to their peers, but that their need to feel accepted and loved by others created a fear of failure and a preoccupation with their performance. Additionally, they were concerned with the way they would feel about their own performance, which may be an internalisation of their belief that people’s worth depends solely on how well they perform. Some were scared to be exposed as imposters. 

### 3.2. Participants’ Pride after Completing the Facilitation Period

After completing the facilitation process, most participants felt a sense of pride. They were proud of having mastered what they initially viewed as threatening tasks: standing in front of the class, creating interest, and working effectively with this age group. One participant who initially had massive self-doubts said she now feels she can achieve ‘most anything’ she aspires to. A facilitator who had experienced failure in a previous teaching role said she felt she had reclaimed a part of herself. 

Participants felt proud when they realised that children internalised the concepts and used communication methods that they had practiced with them. Interestingly, the term ‘pride’ was also used to describe facilitators’ pride in the children, for having managed to share their personal stories and feelings with their classmates. For some facilitators, pride came from acknowledging their impact on specific children:

*I felt proud when I saw that I answered his need to be seen by taking him aside for a personal conversation and explaining his importance to the class and the importance of the program for him. Every time I manage to be precise in my perception of people, I get excited, because I don’t take my success for granted.*

This participant demonstrates a sense of pride in her growing professional abilities. Moreover, it seems that the more profound the self-doubt, the more success was appreciated. Facilitators also experienced pride in their abilities to cooperate well with their co-leader, and to take and give the lead in a harmonious fashion. Additionally, facilitators were proud of their ability to participate in the supervision process and be helped by it. They mentioned being proud of themselves for taking part in various activities they were initially embarrassed by, such as projective exercises. 

Facilitators also emphasised their internal processes as a source of pride, as in Eva’s words:

*Summarizing the process was accompanied by a feeling of relief, a thankfulness for being stuck, for feeling suffocated, for the tears. A door opened in me to discuss my own feelings with myself. I refined my ability to identify the emotion that comes up amongst all the other emotions, and I feel pride in this moment of connection and identification.*

It is interesting and touching to see that Eva is grateful not only for the end result, but also for having been stuck and being able to stay and deal with difficult and sad emotions. She realised that this process is what enabled her to become more attuned with her inner experience and she is proud of that. 

This part will end with going back to Tania, who was ambivalent about her image as ‘the cute fat girl’. At the end of the programme, she said:

*So I went to the school, to the classroom and then something happened inside me, magic started to happen. It was something that I have not experienced for a long time, we had not even started yet, and I already felt really proud of myself.*

It is interesting to note that Tania felt proud even before she began facilitating. From this we can see that her pride emerged not due to performing well, but rather because she was brave enough to try to cope with her fears. 

To conclude, participants felt proud of themselves for performing well or attempting to, despite their great fears. In addition, they experienced pride in their ability to have a good influence on the children and even felt proud of the children. Although these emerging adults are still greatly concerned with their self-worth, at the same time, they felt proud of being able to mentor the younger generation. In addition, they noticed that pride results from external actions: being able to go back into the school, speaking in front of their peers, etc., as well as internal voyages: going deep inside and creating new avenues towards self-understanding and compassion. 

### 3.3. Factors That Supported the Facilitators 

This part explores the components of the programme to which facilitators related their success. Many facilitators mentioned the concepts they have learned and internalised. Whether they spoke of ‘group stages’ or facilitating techniques such as ‘mirroring’, it seems that this acquired knowledge was in part what gave them confidence, helped them perform their facilitation tasks better, and led to their ability to feel proud of themselves:

*Sometimes we were not able to teach the content as smoothly as planned. I presume it depended on the stage of the group. Having studied these stages and experienced them in the great facilitation training programme we received, helped me a lot in coping with the challenges that each stage brought up, in a natural good way.*

In addition to the power of knowledge, this facilitator’s newly acquired confidence is in part due to having received good modelling. 

A highly influential relationship was that of facilitators with their co-leaders. Many of them mentioned ideas, techniques, and overall responsiveness that they learned and adopted from their co-leader. They also spoke about ways in which personal differences between themselves and their co-leader were challenging but helped their self-development:

*We realized that my fears are actually her strengths and vice versa, therefore, I came to the first session with a sense of confidence, knowing we will complement each other.*

Certainly, many participants felt empowered by their co-leader’s supportive approach and appreciation of them (e.g., expressed enthusiasm towards their suggestions, cherished the relationships they have built with the children).

In addition to seeing their supervisors as role models, many facilitators appreciated feeling accepted and validated by these supervisors, who helped them reduce their self-criticism, feel more worthy of love, and more capable of voicing their own opinions. 

Many participants talked about the importance of the peer-group supervision in bringing about change. They felt safe, experienced intimacy, and therefore were able to express themselves freely. The group experience enabled them to better understand the point of view of other people, sometimes the very same people with whose behaviours or opinions they felt most annoyed. In addition, feeling that other students empathised with them after they exposed their greatest fears and vulnerabilities made facilitators feel ‘a sense of warmth’.

*I realized I was not all alone in this world.*

Other facilitators said that their internal, personal work contributed to their success. They mentioned breathing, trying to stay optimistic, looking for their inner voice, and choosing to face their feelings without ‘sanitising them’. Self-acceptance was not only the end result but a part of the individual process they went through:

*I internalized and understood that my differences are my identity, they are what set me apart, it is the force that drives me, not the obstacle that holds me back, as I once thought.*

Finally, facilitators described their contact with the children as personally beneficial in certain aspects. They felt that their ability to be authentic with the children, and even disclose their own straggles when appropriate, helped them. Hearing the children speak of their issues with self-compassion helped facilitators in their own struggle towards self-acceptance. An interesting account comes from Tami who shared that after being able to tell the children that she is still struggling with issues regarding body image, she approached her parents and asked them how she dealt with these issues as a young girl. She also talked with friends who experienced similar conflicts. She said that her current interest is not in ‘closing the cycle’ but rather in opening up her issues and dealing with them with greater awareness.

Considering the entire process reflected in the results, it seems that the facilitators of “Favoring Myself” had significant fears and self-doubts prior to facilitating the programme. In the course of participating in the project, most of them went through a transformational process, ending with a sense of pride in themselves. They related this change to all the resources available to them in the process: the knowledge they acquired, and their connections with important figures such as their peers, co-leader, supervisors, and the children they taught. In addition, they learned to rely on themselves as a source. 

## 4. Discussion

This study explored emotional processes described by student-facilitators of “Favoring Myself”, a wellness school programme. The results showed that although the facilitators had begun their participation in the project quite fearfully, they ended with a sense of pride. This finding is in accordance with studies that described an improvement in confidence and perceived abilities of facilitators [15,16]. This may imply that facilitators experienced an initial lack of confidence in their own abilities, but ended their programmes more self-content. However, previous studies have not reported facilitators’ emotions before and during the training and facilitation processes, and therefore their contributions to understanding the shift experienced by facilitators seem partial. The current study provides knowledge regarding this gap.

This study’s findings show that facilitators seemed quite preoccupied with their performance, striving to be perfect group leaders, and to be seen by others as such. Due to these expectations of themselves they feared failure. Being in their twenties, their fears seem to reflect their stage in life, that is, emerging adulthood. Previous studies showed that emerging adults experience considerable anxieties [24,25] as well as self-doubts and fears of incompetence and failure [27]. A similar study reported that students were worried they would not be able to speak well in public and might face unfavourable audience responses [28]. 

One manifestation of the fear of failure may be the imposter phenomenon. Individuals who perceive themselves as imposters feel less capable than their peers, have difficulty internalising successes, experience a sense of fraudulence, perceive their abilities as overestimated, and believe that others will eventually discover their incompetence. Indeed, some facilitators in the present study described such experiences. This supports previous studies which demonstrated that emerging adults and students in higher education are indeed susceptible to imposter phenomenon [46,47].

Some facilitators in the current study were not only afraid of facilitating children’s groups but were also reluctant to work closely with a co-leader and were worried that they would be judged and not accepted by other facilitators in their peer group. These results support previous research regarding students’ fear of rejection [27]. These findings are also implied by studies demonstrating mentors’ improved sense of social connectedness [15] and relationships [16] following the facilitation experience. Reporting on improvement, facilitators in the latter study indirectly testified that they had experienced initial difficulties or concerns regarding relationships. Facilitators’ social fears could be connected to emerging adults’ social vulnerabilities [26]. 

Furthermore, participants reported various types of fears not revealed in other studies. Some feared they were less competent than their co-leaders, while others feared re-entering the school setting where they experienced a difficult time as children. Some feared repeating dysfunctional social patterns from their childhood. These fears and concerns could all be explained by Arnett’s [24] dimension of ‘negativity’, conceptualising emerging adults’ tendency to be overwhelmed and unsettled. Arnett’s dimension ‘feeling in-between’ could perhaps explain why facilitators were reluctant to teach the children about themes that were still conflictual in their own lives. Emerging adults are neither adolescents nor adults; therefore, while they have a sense of the messages they would like to deliver to the children, they do not yet stand on a steady ground from which they might feel comfortable and authentic when they attempt to communicate these ideas.

After completing the facilitation process, most participants felt proud of themselves for performing well, and even for having the courage to confront their fears by choosing to participate in the project. This echoes previous findings that showed that pride was positively associated with students’ successful performance and achievement [29]. Therefore, emerging adult facilitators’ sense of pride relates to Arnett’s [24] dimension of ‘experimentation’, characterised by optimism. 

Moreover, participants in this study felt proud of their ability to contribute to the children, and were even proud of the children. This form of pride seems to be in line with previous findings regarding mentors’ improved values and sense of belonging to their community [11,12]. These aspects could be connected to Arnett’s [19] dimension of ‘identity’. 

Describing the factors that had assisted them, facilitators mentioned many mechanisms of support available to them in the programme. They felt supported by the knowledge they received directly as well as through modelling from their group supervisors. They internalised moments of success when working as facilitators to raise their self-confidence. They reported receiving help from peers and supervisors. They also spoke about self-reliance and their own internal processes that helped them find meaning in experiencing their difficulties and enduring them. Their abilities to use internal and external resources could be related to the growing capacities of emerging adults, such as the ability to reflect on their environment as well as on their internal states, along with improvements in emotional regulation, problem solving, and ability to compromise [22]. 

According to the self-determination theory [29,30], the various external and mental resources mentioned reinforced growth-oriented processes in the facilitators by fulfilling three psychological needs: relatedness, competence, and autonomy. Relatedness was met by the personal connections created in the programme, which made the facilitators realise they were part of a community in which they felt protected and worthy of love. The need for competence, typically fulfilled when people feel capable of achieving goals and being efficient, was met by facilitators’ experience of success with the children and in their functioning with students of their group. Having faced their various fears, overcoming them by facilitating the program also seemed to have alleviated their sense of competence. The need to take self-directed actions, and exercise freedom of choice fulfilled their sense of autonomy. They were given a great deal of choice as to the ways in which they taught their groups, and as to how they divided the roles between themselves as co-leaders. Autonomy is also met when one experiences pleasure, and indeed facilitators reported they enjoyed the process despite their initial concerns [31]. 

This positive shift can be also seen through the lens of the imposter phenomenon. Gaining relevant experience, avoiding exposure to external comparison and evaluation, and self-validation all contribute to diminished imposter feelings [47]. Indeed, facilitators acquired a great deal of knowledge during the project, which enabled them to perceive their peers as well as professional supervisors as non-judgmental sources of assistance, while many of them attempted to be self-validating and less harsh on themselves. 

Regarding the shift from fear to pride—this discussion raises a dilemma: is this process all-positive? Overcoming fears and developing a sense of pride are certainly important developmental achievements for young individuals. Nevertheless, this transformation relies heavily on the experience of success, and thus it might be part of a never-ending race to perform better and better. This emphasis on performance is not merely a personal issue or tendency; participants’ wish to be perfect, and the resulting reliance on external praise and imposter phenomena, represent social issues pertaining to these facilitators’ generation. 

Arnett emphasised that emerging adulthood is a cultural-sociological phenomenon, present in economically developed countries, in which the age of marriage, parenting, and committing to stable jobs has greatly risen, creating a new stage with its own sense of freedom but also instability and anxieties [23]. A recent study on perfectionism demonstrated that emerging adults are more demanding of themselves. The authors related the phenomenon to the current emphasis on competitive individualism which drives youngsters to attempt to perfect themselves [48].

Arnett saw these processes as not only identifying the zeitgeist of a given generation as it approaches maturity but insisted that the cohort he studied represented a new developmental phase that would be repeated in subsequent generations [49]. If this is so, learning how to help emerging adults actualise themselves and contribute to others at lower personal costs remains a crucial mission. The breadth and depth of training and supervision for the facilitators of “Favoring Myself” helped them go through a challenging process with a great deal of support and care that enabled them to transform from fear to success in a healthy manner. We would like to encourage this generation and the adults supporting them in their studies, volunteer roles, or early occupational positions to help them be fully aware of personal strivings as well as the societal ethos of performance and achievement, and remember to also “Favor Themselves” in their endeavours. 

Regarding the limitations of this study, the size of the sample was quite small and included only students in a particular cultural setting, which may limit representability and generalisability. In addition, the second author, a master’s degree researcher who interviewed the participants was herself a facilitator in the program during the same years, which may have increased social desirability and thus influenced their responses. More qualitative studies need to be performed on mentor and facilitators of prevention programmes in order to enrich knowledge of the ways they are influenced by their participation. 

## 5. Conclusions

Facilitators of “Favoring Myself” had significant fears and self-doubts prior to facilitating the programme. In the course of participating in the project, most of them went through a transformational process, ending with a sense of pride in themselves. They related this shift to support that came from others—peers and professional supervisors, as well as to internal processes. It is the belief of the authors that their fears are typical of current emerging adults. The breadth and depth of training and support that the facilitators received in this project helped make their journey from fear to pride balanced and healthy. It is recommended that other programmes with emerging adults as facilitators be aware of these facilitators’ capabilities and sensitivities, and design their training and ongoing supervision in accordance with emerging adults’ psychological and societal needs. 

## Figures and Tables

**Table 1:** Descriptive characteristics of participants.

	Facilitators
	(n = 33)
Age, M (SD)	26.0 (1.3)
Gender (n, %)	
Female	28 (84.8%)
Male	5 (15.2%)
Major (n, %)	
Education	12 (36.4%)
Nutritional Sciences	21 (63.6%)
Parental marital status	
Married	27 (81.8%)
Separated/Divorced	6 (18.2%)
Socioeconomic status *, M (SD)	0.9 (0.4)

* Calculated by number of people per room in residence. M indicates mean; SD, standard deviation.

## Data Availability

The data that support the findings of this study are available from the second author upon reasonable request and with the permission of The College IRB. The data are not publicly available due to privacy concerns.

## Supplementary Materials

The following supporting information can be downloaded at: https://www.mdpi.com/article/10.3390/ijerph19148421/s1. S1: Thematic interview guide; S2: Instructions for the final essay.
