# Supplementary material for: From Self-Doubt to Pride: Understanding the Empowering Effects of Delivering School-Based Wellness Programmes for Emerging Adult Facilitators—A Qualitative Study
Source: Int J Environ Res Public Health. 2022 Jul 10;19(14):8421. doi: 10.3390/ijerph19148421 (PMC9319596; doi:10.3390/ijerph19148421)
Supplement: Supplementary file 1 [file ijerph-19-08421-s001.zip › ijerph-1714736-supplementary.pdf]

Supplementary Material S1: Thematic interview guide

Thematic semi-structured interview guide: Understanding the Empowering Effects of Delivering School-based Wellness Programmes for Emerging Adult Facilitators

Opening question: Tell me about your experience facilitating "Favoring Myself"

Additional probs were used to ensure that all topics were covered. Each interview was different; however, these were the typical probs:

- How was the preparatory course for you?
- How did you feel prior to beginning the actual facilitation process?
- How was it for you to work with the children?
- How was working with a co-leader for you? How did you divide responsibilities and roles between the two of you?
- What do you think your co-leader have learned from working with you?
- Tell me about the supervision process – individual and with your co-leader.
- How did you experience the peer-group supervision sessions?
- What barriers did you face while delivering the program?
- What had helped you in the process of facilitating "Favoring Myself"?
- What would you suggest should be done differently next year?
- How did you feel at the end of the programme?
- What did you personally gain from delivering the program?

Supplementary Material S2: Instructions for the final essay

<u>Instructions for writing a final essay at the end of the academic course:</u>

1. Background of the setting in which you facilitated "Favoring Myself"; describe the class in general, the pupils, the teacher, atmosphere in the classroom (half a page).

2. Present the programme; what were the goals of "Favoring Myself"? How did you plan to achieve these goals? What facilitating tools did you choose to utilize? What were the challenges you met? How did you cope with them? What were the achievements you experienced? (Up to 2 pages)

3. Personal reflective process; describe your experience facilitating the programme. How did the programme contribute to you individual and professional developmental processes? Please bring examples to clarify your experience. Relate to working with the children, working with the teachers, your peer group experience, cooperating with your co-leader, and your experience in supervision. Use one-two academic papers that can shed light on your experience (Up to 5 pages).
